# Sinonasal analogue HPV related breast multiphenotypic carcinoma, a report of a case with the first description in the breast

**DOI:** 10.1186/s13000-020-01050-7

**Published:** 2020-11-20

**Authors:** Diana M. Oramas, Diana Bell, Lavinia P. Middleton

**Affiliations:** UT MD Anderson Cancer Center, 1515 Holcombe Blvd Box 85, Houston, TX 77030 USA

**Keywords:** Human papillomavirus, Multiphenotypic sinonasal carcinoma, Breast, Basaloid squamous carcinoma, Adenoid cystic carcinoma, Triple-negative breast carcinoma, Basal-like carcinoma

## Abstract

**Background:**

High grade basal-like breast carcinomas are triple negative, express basal cytokeratins, and are known for the overall poor prognosis and aggressive behavior. HPV related multiphenotypic sino-nasal carcinoma has overlapping histology with basal-like breast carcinomas, but carry the defining feature of association with high risk HPV.

**Case presentation:**

We present a case of a perimenopausal woman with a non-healing ulcerated lesion involving the nipple and breast following a trauma. Biopsy performed showed an HPV-positive basal-like carcinoma with squamous differentiation involving the breast, analogous to multiphenotypic carcinoma previously described in the sinonasal tract.

**Conclusion:**

This is the first report of a case of a high- risk HPV related basal-like carcinoma with squamous differentiation, described in the literature. We highlight the morphology and immunophenotype of this lesion and its recognition when compared to other multiphenotypic lesions of the breast, and suggest that pathologists should consider HPV evaluation when encountering similar basal-like tumors involving the breast.

## Background

Basal-like breast carcinomas comprise approximately 15% of all breast cancers. They present usually in young adult, African-American women. Histologically, this tumor shows a heterogeneous morphology with high-grade cytologic features, dense lymphocytic infiltrate, central necrosis and high mitotic index [1–3]. By immunohistochemistry, basal-like breast carcinomas are triple negative, that is negative for ER, PR hormone receptors and HER2, while positive for basal cytokeratins 5/6, 14, 17 and EGFR [3–5]. Their distinction from basaloid salivary gland analogue breast carcinoma, a triple negative carcinoma, with low grade features and good prognosis is imperative for appropriate management due to the aggressive behavior and poor prognosis of basal-like breast carcinomas when compared with basaloid salivary analogy tumors. We present a case of basal-like breast carcinoma, high-risk HPV related, with involvement of the nipple, resembling the HPV-related multiphenotypic sinonasal carcinoma, previously considered an entity specific to the head and neck region.

## Case presentation

**Fig. 1:**
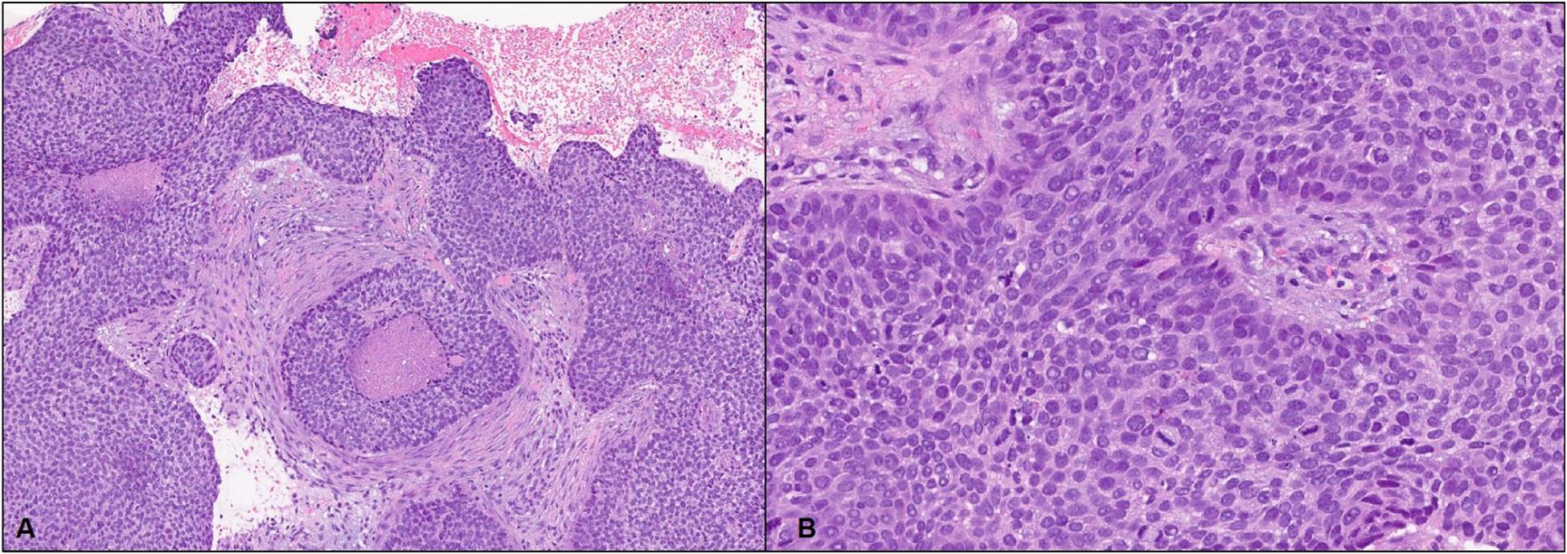
H&E staining showing histologic features. **a**. Low power view shows a basal-like carcinoma infiltrating in nests with desmoplastic background and central necrosis. **b**. High power view shows hyperchromatic palisading spindled cells with nuclear atypia and increased mitotic activity. Central fibrovascular cores are readily identified

**Fig. 2:**
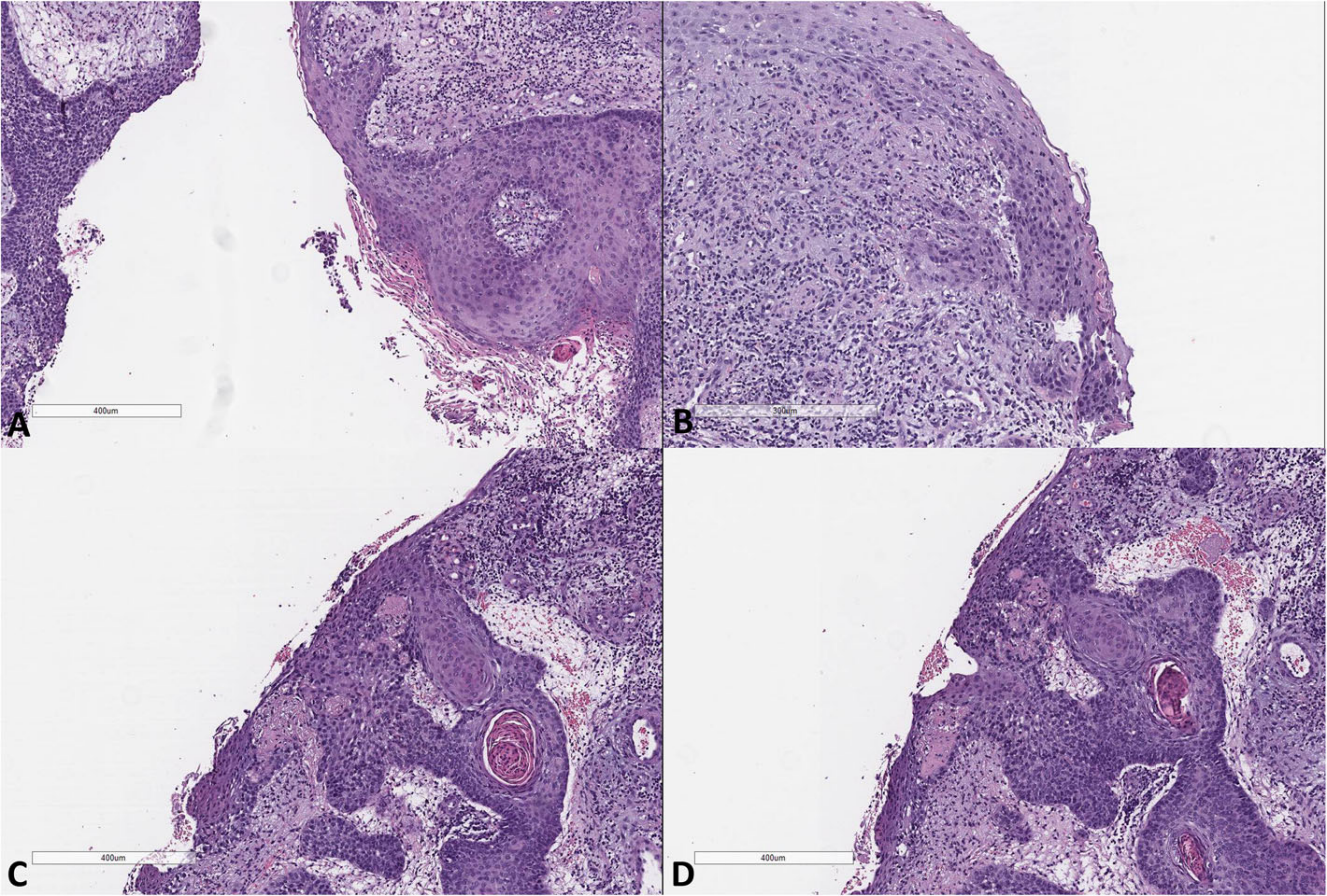
H&E stain demonstrating the variable histological features of the cancer involving the surface epithelium of the nipple (**a**-**d**). The mucosal cells are enlarged, hyperchromatic and dysplastic

**Fig. 3:**
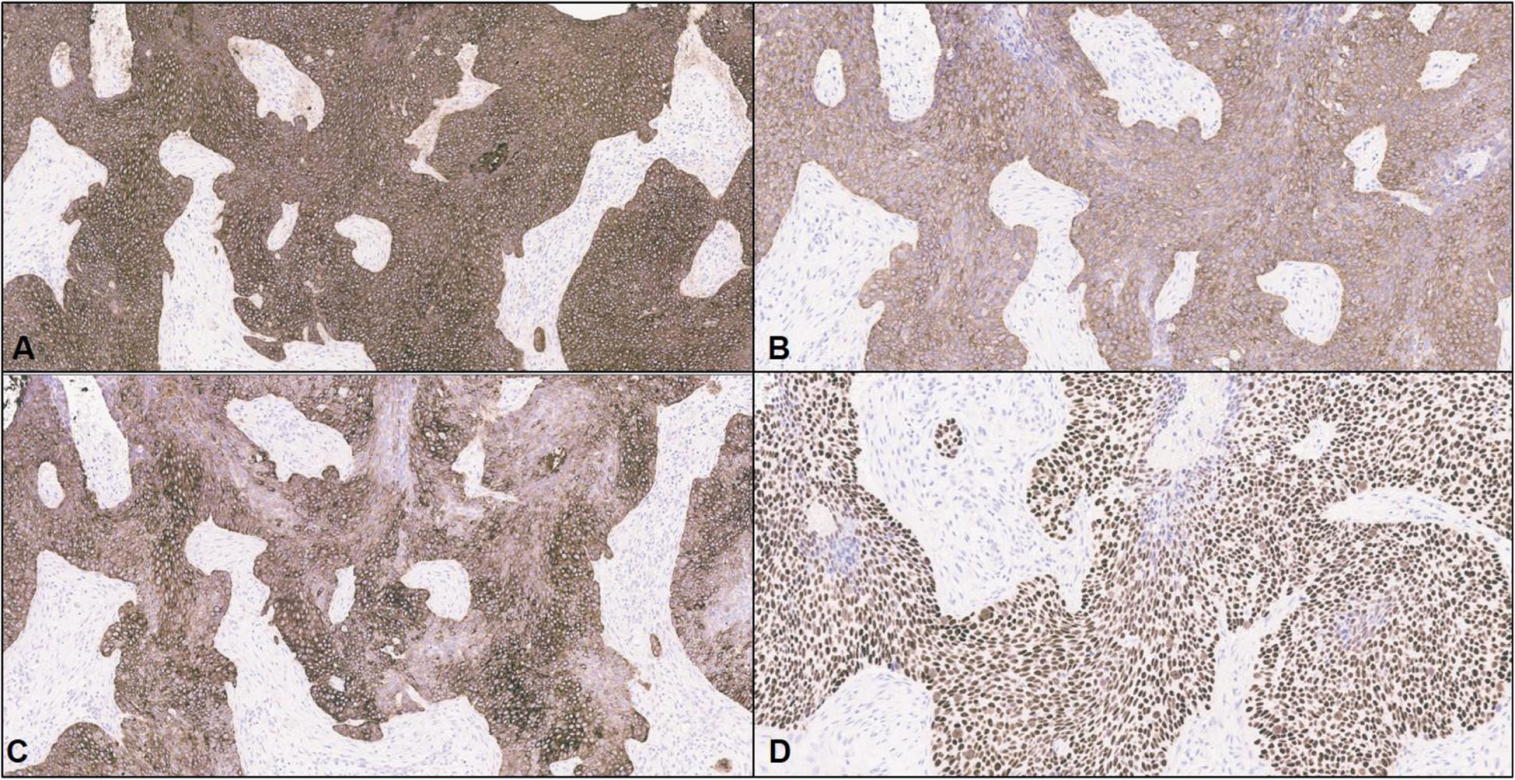
Immunohistochemical studies. Basal cytokeratins: **a**. cytokeratin 5/6, **b**. cytokeratin 14, and **c**. cytokeratin 17 showing strong cytoplasmic and membranous staining. **d**. p63 showing strong nuclear staining, supporting myoepithelial differentiation

**Fig. 4:**
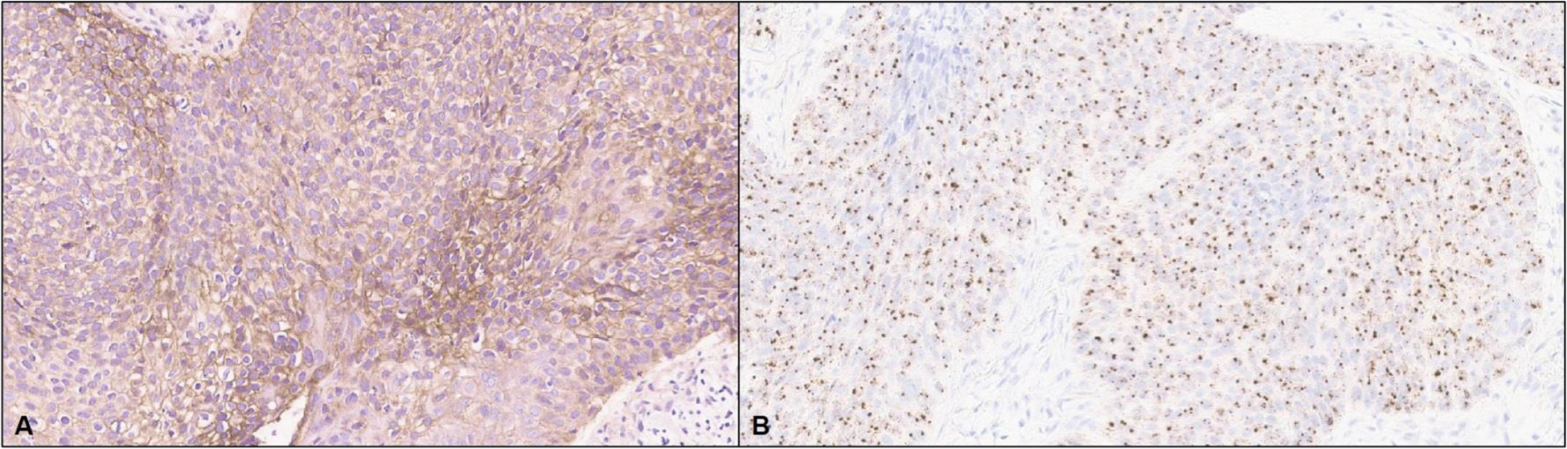
**a**. EGFR by immunohistochemistry showing strong membranous staining. **b**. High risk HPV 33 is positive by in situ hybridization, supporting the diagnosis of HPV-related multiphenotypic carcinoma involving the breast

A 45-year-old perimenopausal woman with no known medical history presented to an outside institution with a nonhealing-ulcerated lesion on the nipple for 6 months following a trauma. On physical examination, a 10 cm fungating ulcerating mass was present involving her left breast, close to the nipple with a 6 cm ipsilateral axillary mass. The rest of the physical exam was unremarkable. Ultrasound findings were consistent with a primary breast carcinoma with axillary lymph node involvement. Histologic examination of the breast and nipple showed features of a high-grade carcinoma with basaloid and squamous phenotypes containing focal areas of comedonecrosis (Fig. 1). The tumor cells exhibited a solid pattern of growth, and expansile pushing borders. The surface epithelium of the nipple was ulcerated with enlarged dysplastic nuclei, dyskeratotic keratinocytes and individual cell necrosis (Fig. 2). Immunohistochemical studies performed on replicate sections show that the tumor cells were positive for GATA3, cytokeratins cocktail, basal cytokeratins: cytokeratin 5/6, cytokeratin 14, cytokeratin 17, as well as p63 (Fig. 3). S100 was negative. Rare cells tumor cells stained for CD 117 (less than 1%) and LEF-1 (5%). EGFR by immunohistochemistry was positive (2+) (Fig. 4). The proliferation index as assessed by Ki67 staining was high with approximately 70% of tumor nuclei staining. Estrogen and progesterone hormone receptors were negative as well as HER2 (score 0). Tumor cells were positive for High-risk Human Papilloma Virus (HPV subtypes tested - 16, 18, 31, 33, 35, 45, 52 and 58) by RNAScope HPV HR8 assay (Fig. 4). Axillary lymph nodes were positive for metastatic disease.

Based on the tumor morphology, immunohistochemical pattern, and HPV positivity, the diagnosis of a HPV related Basal and Squamous Carcinoma involving the breast, analogous to HPV related multiphenotypic sinonasal carcinoma, was rendered.

## Discussion

Herein we present the first case of HPV-related multiphenotypic cancer involving the breast.

Historically, HPV-related cancers were characterized by originating from the oropharynx, harboring non-keratinized squamous histology, and associated with high risk HPV 16 [6]. [7] More recently, HPV related carcinomas have been broadened to include mucosal lesions exposed to the external environment including cervix, anus, vulva, vagina and penis [8]. We postulate that when the mucosal surface of the nipple which contains between 15 and 20 orifices corresponding to the number of subsegmental ducts, is exposed to the external environment, it may be susceptible to HPV related disease. In the head and neck area, where this tumor is more common, HPV-related multiphenotypic sinonasal carcinoma (HMSC) previously described as “HPV-related carcinoma with adenoid cystic carcinoma-like features” has been redefined. In 2013, Bishop et al. first described it as a tumor restricted to the sinonasal tract region [9]. Subsequently, a series of 49 cases of tumor originating in the head and neck region were added to the literature [10]. In this expanded cohort, Bishop concluded that HMSC is truly a distinct histopathologic entity from adenoid cystic carcinoma and we agree. Histologically, HMSC presents as either a high grade keratinized or non-keratinized squamous cell carcinoma with a salivary gland carcinoma component that demonstrates solid nests of basaloid cells with myoepithelial differentiation [9, 11, 12]. The association of high-risk HPV, particularly type 33 was thought to be exclusive of this tumor in the sinonasal tract in the head and neck area [1, 13] [14]. While high risk HPV has been described in other tumors and associated with salivary type adenoid cystic carcinoma (ACC) of the uterine cervix, this entity is very rare [12]. In Bishop’s expanded series of 49 cases, 69% HMSC demonstrated an unusual pattern of surface involvement with markedly atypical cells colonizing the mucosae similar to the current case. All of Bishop’s cases were positive for p16 by immunohistochemistry and HPV by RNA in situ hybridization. Two-thirds of the cases were positive for high risk HPV 33. Variable expression of MYB by immunohistochemistry has been described [15]; however, the *MYB-NFIB* transcripts were not detected in the 49 cases of multiphenotypic sinonasal carcinoma studied by Bishop et al. [10].

Breast carcinomas with basal-like phenotypes have been parallelly described in the literature [1, 2], and core basal phenotype has been described as a subset of triple negative breast cancers that are positive for CK5/6 and EGFR [3]. However, to date, no breast carcinomas have been reported to be associated with high risk HPV. Importantly, the high-risk HPV status of the currently described breast cancer makes this tumor comparable to the features previously described within the histologic spectrum of HMSC involving the head and neck area. Specifically the histology of this triple negative high-grade basal-like carcinoma with squamous differentiation involving the breast has solid nests of basaloid cells with peripheral palisading, an inverted growth patten with expansile nests and central fibrovascular cores. Rare glandular formation is identified. The tumor has a high mitotic rate and frequent mitoses are identified. The surface of the nipple and lactiferous duct showed markedly atypical squamous cells with dyskeratotic keratinocytes and squamous pearl formation, comparable to the previously described HSMC of the head and neck region. The immunohistochemical profile of this tumor consisting of p63 and high weight cytokeratin 5/6, 17 and 14 positivity in the tumor cells along with EGFR immunoreactivity are immunophenotypically compatible with HSMC and a “basal-like breast carcinoma” or “core basal” phenotype as defined by the Neilson Criteria [14]. Cases of HPV-related multiphenotypic carcinoma of the breast resembling HMSC have not previously been reported in the literature. Our case describes the first patient with the a HPV related basal type carcinoma with squamous differentiation, analogous to “multiphenotypic carcinoma” of the sinonasal tract and provides an important rebut to what was previously considered a peculiar variant of head and neck cancer restricted to the sinonasal tract [9]. The outcome and the overall prognosis of this tumor is unknown. The high-grade morphology may warrant a more aggressive treatment and close follow up of these cases. However, more patients will need to be identified and followed in order to define the clinical behavior of this entity. A caveat to remember is that while HMSC usually presents as a large and destructive sinonasal mass with high grade histologic features, it paradoxically behaves in an indolent manner [10].

The differential diagnosis of this tumor includes other basal-like breast cancers. In aggregate, basal-like breast carcinoma comprises a 12–15% of all breast lesions and is characterized for showing a triple negative immunophenotype with the absence of hormone receptors ER, PR and HER2. It is more commonly described in perimenopausal African American women and has an overall poor prognosis. Morphologically the tumor shows a heterogeneous architecture; it can present as high-grade ductal/usual type invasive breast carcinoma with prominent lymphocytic infiltrate and necrosis [13]. The cells are hyperchromatic, often spindled with an increased nuclear to cytoplasmic ratio, vesicular chromatin and prominent nucleoli. Other variants seen are salivary gland-like, medullary-like and metaplastic-like pattern [1, 16]. The salivary gland-like breast carcinoma has been noted to resemble the high-grade solid variant of adenoid cystic carcinoma. The immunophenotype as highlighted before shows a triple negative breast carcinoma with tumor cells demonstrating myoepithelial differentiation. The cells are usually positive for high molecular weight cytokeratins 5/6, 14 and 17 also known as “basal keratins” [4, 5]. EGFR is positive in 75% of cases of basal-like breast carcinoma [4, 17]. Foremost, the basal-like designation of these tumors is based on the expression of basal keratins of the tumor cells rather the morphologic features alone. The differential diagnosis of this tumor is with a basaloid salivary gland analogue tumor of the breast, which in contrast, present as low nuclear grade triple negative carcinoma and subsequently are associated with a better prognosis and require less aggressive treatment recommendations, when compare to the high nuclear grade, histologically aggressive basal-like breast carcinoma.

In conclusion, this is the first case described in the breast with nipple involvement of HPV-related basal-like carcinoma with squamous differentiation, analogous to HPV-related multiphenotypic carcinoma of the sinonasal tract. It is important to recall that the nipple, a mucosal surface exposed to the external environment, can be susceptible to HPV related diseases. The associated high-grade histologic features of this tumor including necrosis, increased number of mitoses and high nuclear grade illustrated herein may guide the pathologist to consider HPV-specific testing when encountering similar “basal-like” lesions in the breast. The histomorphology may portend aggressive behavior when compared morphologically and immunophenotypically to other basal-like tumors of the breast and perhaps the recognition of this entity by other pathologists and communication to clinicians will lead to the development and use of targeted therapy.

## Data Availability

Not applicable.

## References

[1] Gazinska P, Grigoriadis A, Brown JP, Millis RR, Mera A, Gillett CE (2013). Comparison of basal-like triple-negative breast cancer defined by morphology, immunohistochemistry and transcriptional profiles. Mod Pathol.

[2] Badve S, Dabbs DJ, Schnitt SJ, Baehner FL, Decker T, Eusebi V (2011). Basal-like and triple-negative breast cancers: a critical review with an emphasis on the implications for pathologists and oncologists. Mod Pathol.

[3] Cheang MC, Voduc D, Bajdik C, Leung S, McKinney S, Chia SK (2008). Basal-like breast cancer defined by five biomarkers has superior prognostic value than triple-negative phenotype. Clin Cancer Res.

[4] Nielsen TO, Hsu FD, Jensen K, Cheang M, Karaca G, Hu Z (2004). Immunohistochemical and clinical characterization of the basal-like subtype of invasive breast carcinoma. Clin Cancer Res.

[5] P. RP. (2014). Rosen's breast pathology.

[6] Gillison ML, Koch WM, Capone RB, Spafford M, Westra WH, Wu L (2000). Evidence for a causal association between human papillomavirus and a subset of head and neck cancers. J Natl Cancer Inst.

[7] Hwang SJ, Ok S, Lee HM, Lee E, Park IH (2015). Human papillomavirus-related carcinoma with adenoid cystic-like features of the inferior turbinate: a case report. Auris Nasus Larynx.

[8] Pytynia KB, Dahlstrom KR, Sturgis EM (2014). Epidemiology of HPV-associated oropharyngeal cancer. Oral Oncol.

[9] Bishop JA, Ogawa T, Stelow EB, Moskaluk CA, Koch WM, Pai SI (2013). Human papillomavirus-related carcinoma with adenoid cystic-like features: a peculiar variant of head and neck cancer restricted to the sinonasal tract. Am J Surg Pathol.

[10] Bishop JA, Andreasen S, Hang JF, Bullock MJ, Chen TY, Franchi A (2017). HPV-related multiphenotypic Sinonasal carcinoma: an expanded series of 49 cases of the tumor formerly known as HPV-related carcinoma with adenoid cystic carcinoma-like features. Am J Surg Pathol.

[11] Andreasen S, Bishop JA, Hansen TV, Westra WH, Bilde A, von Buchwald C (2017). Human papillomavirus-related carcinoma with adenoid cystic-like features of the sinonasal tract: clinical and morphological characterization of six new cases. Histopathology..

[12] Grayson W, Taylor L, Cooper K (1996). Detection of integrated high risk human papillomavirus in adenoid cystic carcinoma of the uterine cervix. J Clin Pathol.

[13] Boland JM, McPhail ED, García JJ, Lewis JE, Schembri-Wismayer DJ (2012). Detection of human papilloma virus and p16 expression in high-grade adenoid cystic carcinoma of the head and neck. Mod Pathol.

[14] Elgart K, Faden DL (2020). Sinonasal squamous cell carcinoma: etiology, pathogenesis, and the role of human papilloma virus. Curr Otorhinolaryngol Rep.

[15] Shah AA, Oliai BR, Bishop JA (2019). Consistent LEF-1 and MYB Immunohistochemical expression in human papillomavirus-related multiphenotypic Sinonasal carcinoma: a potential diagnostic pitfall. Head Neck Pathol.

[16] Rakha EA, Reis-Filho JS, Ellis IO (2008). Basal-like breast cancer: a critical review. J Clin Oncol.

[17] Walls AL, Middleton LP, El-Naggar AK, Sahin AA. Basaloid Salivary Gland-Analogue Tumors of the Mammary Gland: Clinicopathologic Assessment of a Rare Subtype Intl J Pathol Clin Res 2016;2(042):1–6..

